# Dysregulation of Astrocytic Glutamine Transport in Acute Hyperammonemic Brain Edema

**DOI:** 10.3389/fnins.2022.874750

**Published:** 2022-06-06

**Authors:** Magdalena Zielińska, Jan Albrecht, Mariusz Popek

**Affiliations:** Department of Neurotoxicology, Mossakowski Medical Research Institute-Polish Academy of Sciences, Warsaw, Poland

**Keywords:** glutamine, hyperammonemia, astrocytes, edema, Slc38a3, Slc7a6

## Abstract

Acute liver failure (ALF) impairs ammonia clearance from blood, which gives rise to acute hyperammonemia and increased ammonia accumulation in the brain. Since in brain glutamine synthesis is the only route of ammonia detoxification, hyperammonemia is as a rule associated with increased brain glutamine content (*glutaminosis*) which correlates with and contributes along with ammonia itself to hyperammonemic brain edema-associated with ALF. This review focuses on the effects of hyperammonemia on the two glutamine carriers located in the astrocytic membrane: Slc38a3 (SN1, SNAT3) and Slc7a6 (y + LAT2). We emphasize the contribution of the dysfunction of either of the two carriers to *glutaminosis*- related aspects of brain edema: retention of osmotically obligated water (Slc38a3) and induction of oxidative/nitrosative stress (Slc7a6). The changes in glutamine transport link *glutaminosis-* evoked mitochondrial dysfunction to oxidative-nitrosative stress as formulated in the “Trojan Horse” hypothesis.

## Metabolic Characteristics of Glutamine in Mammalian Tissues: A Brief Account

The presence and roles of glutamine common to all mammalian tissues have been dealt with in many excellent reviews (Häussinger, 1990; Labow and Souba, 2000; Tapiero et al., 2002; Oehler and Roth, 2003; Bak et al., 2006; Hakvoort et al., 2017), and therefore are mentioned here only briefly. Glutamine is the most abundant free amino acid and accounts for ∼50% of the total free amino acids in the human body (Spodenkiewicz et al., 2016). In peripheral tissues, glutamine is most abundantly represented in muscles (more than 40% of the free amino acid pool) and blood plasma (more than 20%, concentrations ranging from 0.4 to 0.7 mM) (Bergström et al., 1974; Kuhn et al., 1999). Glutamine participates in a variety of metabolic pathways, thereby controlling an abundance of biological processes. Ubiquitously, it serves as a precursor of purines and pyrimidines, is involved in the synthesis of amino sugars, regulation of pH, glutathione homeostasis, energy production, etc. (Cory and Cory, 2006; Albrecht et al., 2010). Systemically, enhanced glutamine synthesis exerts beneficial effects on the immune system (Castell et al., 2004) or gut barrier (Krishna Rao, 2012; Wang et al., 2015). Glutamine also stimulates branched-chain amino acids catabolism in skeletal muscle (Mann et al., 2021).

## Glutamine Functioning in the Brain Depends on Its Mobility

In the mammalian brain, glutamine is a conditionally essential amino acid, synthesized from glutamate and ammonia, in a reaction mediated by glutamine synthetase (GS), an enzyme overwhelmingly (Martinez-Hernandez et al., 1977), albeit not exclusively (Amaral et al., 2013), located in astrocytes. Astrocytic glutamine is a precursor of the releasable pool of the excitatory neurotransmitter amino acid glutamate and the inhibitory amino acid, γ-aminobutyric acid (GABA), a sequence of reactions characterized by rapid cellular turnover rates (Bak et al., 2006; Rose et al., 2013). Glutamine released by astrocytes is taken up by neurons. In the mitochondria of neurons, glutamine is transformed by phosphate-activated glutaminase (PAG) into glutamate, released as a neurotransmitter into the synaptic space, from where it is taken up by astrocytes, and conjugates with ammonia to form glutamine, coined the glutamine/glutamate cycle (GGC) (see reviews by Yudkoff et al., 2000; Rothman et al., 2003; Bak et al., 2006; Albrecht et al., 2010). Of note, a significant portion glutamate is not converted to glutamine, but is taken up by astrocytes undergoes oxidation subsequent to its conversion to TCA cycle intermediates (McKenna et al., 2016). A portion of glutamine not utilized in GGC leaves the brain *via* the blood-brain barrier (Hawkins and Viña, 2016; Zaragozá, 2020) or blood-CSF barrier (Dolgodilina et al., 2020), by a mechanism involving exchange with large neutral amino acids leucine or tryptophan (Mans et al., 1982; del Pino et al., 1995).

Transport of glutamine within GGC involves transporter members belonging to A and N transport systems that differ in their properties (Bröer, 2007; Leke and Schousboe, 2016). Of note, the issue of the specific, GGC-related roles of glutamine will not be dealt with here, as the effects of hyperammonemia-related excess of glutamine on amino acidergic neurotransmission have so far escaped conclusive elaboration. Here we focus on the implications of the involvement of glutamine in brain ammonia turnover. Because in the brain the urea cycle is incomplete, glutamine synthesis remains the only efficient route of ammonia neutralization and, along with glutamine efflux from the brain to blood, constitutes the critical mechanism for excreting excess ammonia (Cooper and Jeitner, 2016). Since an overwhelming proportion (up to 80%) of astrocytic glutamate is converted to glutamine, glutamate uptake is a critical factor in regulation of glutamine efflux (McKenna et al., 1996, 2016). In line with the above, under controlled ammonia load, glutamine transporters localized on the astrocytic side suffice with facilitated diffusion controlled by the intracellular concentration of glutamine. However, in the setting of ALF, or of acute hyperammonemia evoked by other causes, increased glutamine accumulation resulting from ammonia overload *per se* induces astrocytic swelling, which leads to the often-mortal cytotoxic brain edema. This issue will form a specific theme of the review.

Both ammonia detoxification and the GGC put high demands on the availability of glutamine at its destination loci and rapid escape from its locus of synthesis (Daikhin and Yudkoff, 2000; Schousboe et al., 2014). As discussed in further sections of this review, the escape routes are critical for preventing excessive, edema-inducing accumulation in astrocytes under conditions of ammonia overload. Intercellular mobility of glutamine is facilitated, by its extraordinary abundance in the extracellular space. While brain tissue glutamine concentrations are more or less equal to other amino acids, its concentrations in the extracellular fluid or the cerebrospinal fluid (∼0.5–1 mM) exceeds by one order of magnitude other amino acids in these compartments (Perry et al., 1975; Jacobson et al., 1985; Xu et al., 1998). Most importantly, glutamine mobility is secured by active, highly controlled transport between different cell types of the CNS, at the brain-blood and blood-CSF barrier, executed by glutamine transporters.

## Cell Membrane Glutamine Transporting Systems

Glutamine membrane transporters belong to different protein families that differ in properties, and most of them also accept other neutral or cationic amino acids (Albrecht and Zielińska, 2019). Glutamine transporters share an affinity toward glutamine but show differences in transport modes concerning Na^+^ or H^+^ coupling. The cotransporters efficiently accumulate glutamine while antiporters determine the balance between glutamine and other amino acids. The most acknowledged glutamine transporters belong to the Slc1, 6, 7, and 38 families (Pochini et al., 2014). The pleiotropic role of glutamine may be a reason why glutamine transporters, belonging to several protein families, are both redundant and ubiquitous. In general, none of the proteins is unequivocally specific (Pochini et al., 2014). Glutamine transporter classification originally was based on functional properties, such as substrate specificity, ion and pH dependence, kinetics, and regulatory properties, and led to clustering the transporters in systems. In the mammalian brain, glutamine transport systems are classified into two distinct groups termed sodium-dependent (systems A, ASC, and N) and sodium-independent (system L and y + L). System A and system L, the first defined, indicate carriers’ “alanine-preference” and “leucine-preference,” respectively (Kanai and Endou, 2001; Solbu, 2010). The systems include one or even more transporters, still not identified as specific proteins, due to the difficulties in discriminating a single function in entire cells. Neither system A nor system N-mediated transport requires counter-transport of other amino acids and determines the actual net amino acid flux (Meier, 2002). Table 1 presents astrocyte-localized glutamine transporting moieties with their transport features and, so far identified, functional importance.

**TABLE 1:** Classification and characteristics of astrocytic glutamine transporters.

Family/System/Isoforms			Substrates	Transport mode	Cell type location	References	Meta-analysis of RNA in human brain *	Function/ clinical significance	References
Slc1 (A5) ASC ASCT2			Gln, Ala, Cys, Ser, Thr	Bidirectional/Na^+^ dependent Gln/neutral AA antiport	Cultured astrocytes C6 glioma cell line Müller glia Synaptosomes (Few data)	Bröer et al., 1999; O’Brien et al., 2005; Gliddon et al., 2009	Microglia Oligo-dendrocytes Inhibitory neurons	Amino acids pools regulation Tumors progression	Gliddon et al., 2009; Scalise et al., 2018b
Slc6 (A14) B^(0,+)^ ATB^(0,+)^			Gln, Ser, Gly, Met	Inward 2Na^+^ – 1Cl^–^ Gln cotransport	Astrocytes Capillary endothelial cells	Sloan and Mager, 1999; Czeredys et al., 2008; Samluk et al., 2010; Michalec et al., 2014		Pancreatic cancer Prognostic factor Breast cancer ER + *Slc6a14^–/–^* mice develop obesity, fatty liver, and metabolic syndrome under high-fat diet	Coothankandaswamy et al., 2016; Nałęcz, 2020; Sivaprakasam et al., 2021
Slc7	y + L y + LAT2		Gln, Arg	Bidirectional (outward preferred) Gln/Na + cotransport Arg antiport	Astrocytes Neurons Cultured capillary endothelial cells Much less so *in situ*	Wagner et al., 2001; Lyck et al., 2009	All brain cells microglia (enhanced)	Arg release	Pfeiffer et al., 1998; Bröer et al., 2000
	L	LAT1	Gln, Leu, Val, Met, His, Ile, Tyr, Trp, Phe	Outward Gln/large neutral AA antiport	Endothelial cells Neurons Astrocytes BV2 microglia cell line	Fotiadis et al., 2013; Scalise et al., 2018a; Albrecht and Zielińska, 2019; Huttunen et al., 2019	Microglia	Tumors growth – mTOR pathway Thyroid hormones transport AA-like drugs transport L-DOPA, AA transport Linked to PD Mutations linked with autism spectrum disorder	Kageyama et al., 2000; Nicklin et al., 2009; Bröer et al., 2016; Tǎrlungeanu et al., 2016
		LAT2	Gln, Leu, Val, Met, His, Ile, Tyr, Trp, Phe, Thr, Asn, Cys, Ser, Ala	Outward Gln/neutral AA antiport	Microglia Astrocytes Neurons	Braun et al., 2011	Inhibitory excitatory neurons, microglial cells	Pancreatic cancer neuroendocrine tumors Pro-oncogenic factor	Wang and Holst, 2015; Barollo et al., 2016; Feng et al., 2018
Slc38	N	SN1/SNAT3	Gln, His, Asn	Bidirectional (Outward preferred) Gln/Na^+^ cotransport H^+^ antiport	Astrocytes Müller glia cells Capillary endothelial cells	Chaudhry et al., 1999; Chaudhry, 2001; Boulland et al., 2002; Rubio-Aliaga and Wagner, 2016	Astrocytes Microglia Oligo-dendrocyte precursors Oligodendrocytes	Neurotransmitter synthesis Gluconeogenesis Insulin metabolism mTORC1/SK6 pathway Acid-base balance maintain Squamous carcinoma Non- small cell lung cancer development	Chaudhry et al., 1999; Boulland et al., 2002, 2002; Nissen-Meyer and Chaudhry, 2013; Rubio-Aliaga and Wagner, 2016; Wang et al., 2017; Liu et al., 2020
		SN2/SNAT5	Gln, His, Asn	Bidirectional (Outward preferred) Gln/Na^+^ cotransport H^+^ antiport	Astrocytes	Cubelos et al., 2005	microglia	Glycine release for NMDA receptor regulation Tumor development (unconfirmed) c-Myc target	Wise et al., 2008; Hamdani et al., 2012; Bhutia et al., 2015

*Data from https://www.proteinatlas.org/, ranked from the one with the highest expression.

## Glutamine Cell Membrane Transporters in Focus of This Review

We specifically focus in this review on the ammonia-induced changes in the operation of astrocytic glutamine transporters that, with the evidence presented, have a contributing impact on the development of brain edema in the setting of ALF. Among the glutamine transporting moieties present on the membranes of astrocytes (Table 1), two, according to current data (Table 2), have attracted attention in this context. One of these, Slc38a3, is responsible for effective egress of newly synthesized glutamine from astrocytes and, this is well suited to prevent its excessive intra-astrocytic accumulation. The other one, Slc7A6, promotes glutamine exchange for essential cationic amino acids including, arginine (Bröer et al., 2000).

**TABLE 2:** ALF or ammonia *in vitro*- induced alterations in astrocyte-localized glutamine transporters.

Isoform	Animal model	mRNA	Protein	References	*In vitro* model	mRNA	Protein	Transport	References
y + LAT2	TAA-induced ALF rats	↓	–	Zielińska et al., 2014	Cortical rat astrocytes 48 h with 5 mM NH_4_^+^	↑	↑	L-[^3^H]-arginine uptake ↑	Zielińska et al., 2012, 2015; Milewski et al., 2017
	Ammonium acetate-induced HA rats	Not tested	↑	Zielińska et al., 2011					
SN1/ SNAT3	AOM-induced ALF mice	↓	↓	Hamdani et al., 2021	HEK cells transfected 0.5 or 2 h with 2 or 5 mM NH_4_^+^	–	↓	Not Tested	Hamdani et al., 2021
	TAA-induced ALF rats	↓	↓	Zielińska et al., 2014	PS120 cells transfected 0.5h with 5 mM NH_4_^+^	Not tested	Not tested	L-[^3^H]-glutamine efflux and uptake ↓	Hamdani et al., 2021
	Ammonium acetate-induced HA rats	–	–	Zielińska et al., 2014	Cortical mice astrocytes 24 h with 5 mM NH_4_^+^	–	–	Not tested	Dąbrowska and Zielińska, 2019
	High ammonia-diet-induced HA rats	Not tested	↑	Cabrera-Pastor et al., 2019					
	BDL rats	–	Not tested	Leke et al., 2015					
SN2/ SNAT5	TAA-induced ALF rats	↓	–	Zielińska et al., 2014	Cortical mice astrocytes 24 h with 5 mM NH_4_^+^	–	Not tested	Not tested	Zielińska et al., 2016
	Ammonium acetate-induced HA rats	–	–	Zielińska et al., 2014	PS120 cells transfected 0.5h with 5 mM NH_4_^+^	Not tested	Not tested	L-[^3^H]-glutamine efflux and uptake ↓	Hamdani et al., 2021
	BDL rats	↑	–	Leke et al., 2015					
	Hepatic devasculariz ation in rats	↓	Not tested	Desjardins et al., 2012					
	TAA-induced ALF mice	Not tested	–	Rama Rao and Norenberg, 2014					

*↑, upregulation; ↓, downregulation; “–” – no changes.*

*AOM, azoxymethane; BDL, bile duct ligation; HA, hyperammonemia; TAA, thioacetamide.*

## Glutamine Transport in Astrocytic Mitochondria

Intracellular transport from cytoplasm to mitochondria is one other factor contributing to intracellular (especially intra-astrocytic) glutamine homeostasis. It was demonstrated before that glutamine enters through the mitochondrial outer membrane (Zalman et al., 1980) and passes through the inner mitochondrial membrane using the mitochondrial glutamine carrier (Matés et al., 2009). The transport has been characterized in peripheral mammalian tissues e.g., kidneys (Indiveri et al., 1998), liver (Kovacević et al., 1970; Kovacević and Bajin, 1982; Haussinger et al., 1985), and tumor cells (Molina et al., 1995). Recently, the SLC1A5 carrier variant transporting glutamine has been characterized in mitochondria of cancer cells (Yoo et al., 2020). Although its in-depth characterization in brain mitochondria is pending, there is firm evidence of a saturable, carrier-mediated transport of glutamine in both synaptic and non-synaptic, astrocytic mitochondria (Minn, 1982; Steib et al., 1986; Roberg, 1995; Dolińska et al., 1996). However, the molecular identity of mitochondrial glutamine transporter in control brain tissue has not yet been definitively revealed. Most interestingly glutamine uptake to mitochondrial fractions enriched in large, astrocytic mitochondria is strongly inhibited by histidine (Albrecht et al., 2000; Ziemińska et al., 2000a), and is coupled to its intra-mitochondrial degradation to glutamate and ammonia by PAG (Albrecht et al., 2000; Ziemińska et al., 2004).

## Glutaminosis: A Consistent Neuropathological Component Of Hyperammonemic Encephalopathies

Hyperammonemia defines metabolic disorders characterized by elevated levels of ammonia in the blood. Hyperammonemia occurs in a course of many and often causally unrelated diseases, such as chronic and acute encephalopathy, Reye’s syndrome, congenital deficits in urea cycle enzymes (UCDs), uremic encephalopathy, diabetic encephalopathy, or hypoglycemic encephalopathy.

Typically and most frequently, hyperammonemia is a secondary complication of primary liver disease, a leading factor in hepatic encephalopathy (HE). HE is a multi-symptomatic neurological syndrome related to acute (drug-induced) or chronic (cirrhosis associated) liver failure, where blood-derived ammonia (Ong et al., 2003; Jayakumar and Norenberg, 2018), and blood-derived and endogenous inflammatory intermediates, are the key pathogens (Rodrigo et al., 2010; Coltart et al., 2013; Balzano et al., 2020).

By definition, in hyperammonemic encephalopathies circulating ammonia concentration is significantly increased, reaching high micromolar or even millimolar concentrations in UCDs (Michalak et al., 1996) and ALF patients (Clemmesen et al., 1999). However, biochemical analysis of these patients’ brain tissue, neuroimaging (Kreis et al., 1990; Binesh et al., 2005) and brain microdialysis (Bjerring et al., 2008) showed significantly increased cerebral glutamine concentrations, consistent with the conversion of ammonia into glutamine. While, this did not *per se* prove the pathogenic role of glutamine, more recent studies do. In patients with acute-upon-chronic liver failure, a condition where stable liver cirrhosis undergoes instant exacerbation, the severity of clinical symptoms correlated well with brain glutamine as quantitated by NMR (Laubenberger et al., 1997). In line with the above, in patients with hepatic coma awaiting liver transplantation, the brain’s extracellular level of glutamine measured by microdialysis correlated as faithfully as arterial blood ammonia with intracranial pressure (Tofteng et al., 2006). Of note, increased glutamine content in the extracellular space content is thought to faithfully reflect the excess of glutamine that leaves astrocytes (Bosman et al., 1992; Rao et al., 1995; Hilgier et al., 1999). Notably, the high activity of GS and efficient incorporation of ammonia into glutamine result in a large ammonia concentration gradient between the brain and blood, which, together with the pH difference between the brain (∼7.1) and blood (∼7.4), promotes the penetration of ammonia into the brain (Ott and Larsen, 2004).

## Brain Edema: The Ultimate Phase of Acute Hyperammonemia

The most life-threatening complication associated with acute HE is cerebral edema, in extreme cases resulting in intracranial hypertension severe enough to cause a patient’s death by brain herniation (reviewed in Blei, 2007; Bosoi and Rose, 2013; Scott, 2013). It has been documented that in ALF patients, intracranial pressure positively correlates with arterial ammonia levels (Bernal et al., 2007). The cerebral edema development in ALF progresses quickly and remains the leading cause of high patient mortality (∼50–80% of deaths). To this date, no specific treatment of acute HE-associated brain edema and coma other than whole-body cooling is available (Vaquero and Blei, 2004).

Histopathologic evaluation of ALF patient’s brain tissue revealed characteristic features of brain edema encompassing alterations in the morphology of astrocyte and endothelial cells (Martinez, 1968; Norenberg, 1977; Kato et al., 1992). Increased number of vacuoles and thickened basement membrane were visible in the endothelial cells whereas, swollen astrocytes with swollen mitochondria in the cytoplasm, and enlarged basal membrane, were observed in regard to the astrocyte cell population (Martinez, 1968; Norenberg, 1977; Kato et al., 1992). Of note in this context, characteristic pathomorphologic features of astrocytes, with swollen nuclei, glycogen deposition, and margination of chromatin, known as Alzheimer type 2 astrocytosis, are observed irrespective of the duration of hyperammonemia and its origin (inherited vs. acquired) (Norenberg, 1987). Moreover, imaging studies based on T2-weighted MRI scans in patients with ALF displayed decreased value of an apparent diffusion coefficient, indicating an increase in intracellular water in the brain and the cytotoxic nature of brain edema in ALF (Chavarria et al., 2010). Morphological (Traber et al., 1987, 1989; Kato et al., 1989; Chadipiralla et al., 2012) and MRI-derived evidence obtained in animal models of ALF (Obara-Michlewska et al., 2018; Hamdani et al., 2021) corroborated with observations on patients, further supporting the cytotoxic mechanism of ALF-induced brain edema.

## Acute Hyperammonemic Brain Edema Is Primarily Cytotoxic in Nature

By analogy to other edema-associated brain pathologies, the relative roles of cytotoxic and vasogenic factors in the development of brain edema associated with acute HE has long remained a matter of debate (Blei, 2007). In agreement with the long-held view that HE is primarily an “astrogliopathy” (Norenberg, 1987; Albrecht et al., 2010), current evidence supports its primarily cytotoxic nature, primarily reflecting swelling of astrocytes by mechanisms related to intracellular metabolic and ion imbalance and the ensuing intracellular accumulation of water (Haussinger and Schliess, 2008; Bémeur et al., 2016). The current view of edema pathophysiology, is that ammonia induces astrocytic swelling by a complex interplay of (i) impairment of in- and out-transport of different osmolytes leading to intracellular osmotic imbalance, (ii) mitochondrial dysfunction related to excessive accumulation of ammonia-derived glutamine and successive intra-mitochondrial release of toxic levels of ammonia, (iii) oxidative/nitrosative stress. Evidence presented below strongly suggests that glutamine is involved in a vicious cycle of (i), (ii), and (iii).

## Glutaminosis: A Causative Factor In Cytotoxic Brain Edema And Its Mechanistic Basis

As mentioned in the section: “*Glutaminosis*: a consistent neuropathological component of hyperammonemic encephalopathies,” clinical studies revealed a good correlation between brain glutamine content and the severity of edema-related manifestations of ALF. While correlation strongly suggested a mechanistic link, it could not be interpreted as causal nexus. A proof of concept came from experimental ALF or acute-upon-chronic liver failure models, inhibition of GS activity by a GS inhibitor, methionine sulfoximine, alleviated astrocytic swelling, brain edema, and a set of other pathophysiological symptoms of acute HE (Takahashi et al., 1991; Willard-Mack et al., 1996; Master et al., 1999; Tanigami et al., 2005). Contrary to efficient ammonia-lowering treatments, which are effective in reducing brain edema in animal models of ALF, a decrease in high cerebral glutamine levels were not observed (Zwingmann et al., 2004; Ytrebø et al., 2009).

## Slc38A3: The Transporter Involved in Glutamine Trapping Within Astrocytes

Studies *in vitro* on primary astrocyte cultures indicated that the contribution of system N in glutamine transport varies from 10 to 50% of the total glutamine transport capacity (Nagaraja and Brookes, 1996; Deitmer et al., 2003; Heckel et al., 2003; Dolińska et al., 2004; Sidoryk-Węgrzynowicz et al., 2009). System N silencing in neonatal astrocytes inhibited glutamine efflux out of the cells and documented its prevailing functioning in the direction of glutamine release (Zielińska et al., 2016). Glutamine efflux is preferably mediated by the Slc38a3 system N glutamine transporter, much more so than by one other system N transporter Slc38a2, as the latter shows relatively high affinity for serine, alanine, and glycine (Nakanishi et al., 2001a,b; Hamdani et al., 2012). Immunohistochemical data revealed localization of the system N transporters Slc38a3 (Boulland et al., 2002) and Slc38a5 (Cubelos et al., 2005) on astrocytic processes located adjacent to glutamatergic and GABAergic synapses, confirming the presence of the protein in native tissue. More recently, Slc38a5 was shown to be located intracellularly (Hamdani et al., 2012), and functional expression of Slcs8a3 was demonstrated in the astrocytic plasma membrane *in situ* (Todd et al., 2017). Hence, over thirty years after its initial discovery, it has become clear that Slc38a3 plays a critical role in regulating the astrocytic leg of GGC.

Evidence implicates Slc38a3 transporter to act as a hub for glutamine transport, recruiting a range of accessory complexes that enhance and regulate its function in response to different protein (Boulland et al., 2002; Rubio-Aliaga and Wagner, 2016). Interestingly enough, the physical coupling of Slc1a3-Slc38a3 proteins demonstrated in cultured Bergmann glia (Martínez-Lozada et al., 2013) and kinetics data of glutamine fluxes driven mainly by the increased astrocytic [Na^+^] (Todd et al., 2017) support Slc38a3-mediated glutamine transport in glutamate recycling. Since Slc38a3 preferentially controls the efflux of newly synthesized glutamine from astrocytes (Chaudhry et al., 1999), it prevents excessive accumulation in there and, subsequently, retention of osmotically obligated water. Accordingly, its decreased activity in ALF leads to enhanced intracellular glutamine trapping, in this way contributing to cytotoxic brain edema (Hamdani et al., 2021).

Kanamori and Ross (2005) were the first to hint, albeit indirectly, at the possibility that system N-mediated glutamine transport may be controlled by hyperammonemia. In their study, continuous infusion of [^15^N]ammonium acetate resulted increased glutamine accumulation in brain microdialysate. Since extracellular glutamine sufficiently saturated system A-mediated glutamine transport, the authors interpreted their finding as reflecting inhibition of glutamine efflux from astrocytes by reversal of system N transport. Our recent studies extended this original finding and directly implicated the involvement Slc38a3 in the sequence of events leading to astrocytic swelling and brain edema. Decreased Slc38a3 expression was recorded in the cerebral cortex of rats with thioacetamide-induced ALF, but not in those with ammonium-acetate-induced simple hyperammonemia (Zielińska et al., 2014). Most interestingly, thioacetamide-induced ALF, but not hyperammonemia was earlier found associated with increased cerebro-cortical volume (Hilgier et al., 1996). In azoxymethan-induced mice model of ALF, decreased expression of Slc38a3 in the brain coincided with (i) excessive brain glutamine (ii) increased astrocytic cell volume and (iii) decreased apparent diffusion coefficient marking cytotoxic brain edema (Hamdani et al., 2021). As a proof of concept, local Slc38a3 silencing using *vivo morpholino* technique resulted in astrocytic swelling and increased volume of the brain region subjected to silencing (Hamdani et al., 2021).

The mechanism underlying reduction of Slc38a3 expression in the ammonia- (or otherwise glutamine)- overexposed brain may be subject to regulation by a wide spectrum of transcriptional, translational, or epigenetic factor which remain to be analyzed in detail. Ammonia treatment *in vitro* strongly inhibited the release of newly loaded radiolabeled glutamine from cultured astrocytes (Dąbrowska et al., 2018) and the inhibition involved changes in protein kinase C signaling (Dąbrowska et al., 2018), Sp1-Nrf2 transcription factor complex formation (Dąbrowska and Zielińska, 2019), and activation of Nrf2 transcription factor *per se* (Dąbrowska et al., 2021). Of note in this context, the study by Lister et al. (2018) identified Nrf2 transcription factor control in the regulation of Slc38a3mRNA expression (Lister et al., 2018). In addition, *in vitro* experiments on Slc38a3 transfected oocytes revealed that depression Slc38a3-mediated glutamine transport engages direct interaction of ammonium ions with the Slc38a3 protein moiety (Hamdani et al., 2021). Clearly, the mechanisms underlying the responses of Slc38a3 to ammonia at the transcriptional/translational/posttranslational levels deserve further investigation.

Slc38a3 carrier is believed to bridge inactivation of glutamate in astrocytes to neurotransmitter glutamate synthesis in the nerve endings (Todd et al., 2017; Verkhratsky et al., 2021). Therefore, its excessive involvement in glutamine trapping may contribute to the imbalance between excitatory and inhibitory neurotransmission as well. However, proper glutamatergic transmission seems to be less dependent and may occur after inhibition or reduction of GS (Liang et al., 2006; Kam and Nicoll, 2007) or after local knock out-induced reduction of astrocytic Slc38a3 transporter, mediating glutamine efflux (Popek et al., 2020). It remains to be seen whether structural and electrophysiological impairment of glutamatergic synapse noted in ALF mice is in anyway related to Slc38a3 depletion in the same model (Popek et al., 2018).

## Glutamine: An Osmotic Stress- Inducing Factor

Cerebral glutamine concentration above 2 mM saturates active glutamine transport out of the brain (O’Kane and Hawkins, 2003) and, thereby, favors its accumulation. Accumulation of synthesized in glial cells glutamine, might cause the osmotic pressure increase and generate glutamine concentration gradient leading to the development of intracranial pressure (ICP) (Master et al., 1999; Tofteng et al., 2002, 2006). In the brain, the difference between 1 mmol/L in blood and 4 mmol/L would cause a pressure gradient of 35 mmHg that is well above the critical level to cause brain edema. Thus, simultaneously triggered brain compensatory mechanisms are critical and encompass a removal from the brain other osmotically active compounds, e.g., myo-inositol, choline, or taurine, which at least partially reduce brain osmotic effects (Sterns and Silver, 2006; Heins and Zwingmann, 2010; Jiménez et al., 2017). Importantly, biochemical changes of different osmotically active substances in the brain are frequently observed even before the onset of clinical symptoms (Larsen, 2002). Studies performed by proton magnetic resonance spectroscopy (^1^H MRS) in symptomatic patients with severe ornithine transcarbamylase deficiency showed that a significantly elevated level of glutamine was accompanied by a simultaneous decrease in myo-inositol and choline, which is considered compensatory in nature (Gropman et al., 2009; Sen et al., 2021). However, at a very high concentration of ammonia, osmoregulation may be insufficient to compensate for the rapid cerebral glutamine increase. The above is supported by the results delivered from animal models: as outlined in the previous section, brain edema caused by acute ammonia poisoning was prevented by the inhibition of glutamine synthesis. However, arguments may be raised against the contribution of excess glutamine to increased intracellular osmotic pressure. Mild hypothermia prevented brain edema development although, glutamine brain content remained relatively stable under these conditions (Vaquero and Butterworth, 2007; Stravitz and Larsen, 2009). Moreover, in the healthy brain glutamine accounts for less than 2% of the total pool of organic osmolytes and electrolytes (Pasantes-Morales et al., 2002). Therefore, the ∼3–4-fold increase of brain glutamine observed in ALF patients (Bjerring et al., 2010), and experimental animals (Takahashi et al., 1991; Master et al., 1999), is unlikely to be enough to significantly account for brain edema, even if not compensated by the efflux of other osmolytes. Of note, water flow through the cell membrane is at least in part regulated by the activity of aquaporin water channels which additionally may modify the cell’s osmotic status (Papadopoulos and Verkman, 2007; Thrane et al., 2011). All in all, although perhaps significant locally, on a global scale, the osmotic effect of excess glutamine alone is unlikely to be the predominating edema-eliciting factor.

## Glutamine: Inducer of Mitochondrial Dysfunction and Swelling in the “Trojan Horse” Hypothesis

According to the “Trojan Horse” hypothesis (Albrecht and Norenberg, 2006), glutamine accumulating in excess in the cytoplasm enters mitochondria and opens the megachannel in the inner mitochondrial membrane, mitochondrial permeability transition pore (mPTP), which is sensitive to cyclosporine A (Ziemińska et al., 2000b). Available evidence suggests that glutamine enters mitochondria by active transport rather than by diffusion. It was shown that glutamine entry to astrocytic mitochondria is stimulated by pathopysiologically relevant concentrations of glutamine (Dolińska et al., 1996). Histidine, the strongest inhibitor of mitochondrial glutamine among all the amino acids (see section “Glutamine Transport in Astrocytic Mitochondria”), attenuated opening of mPTP and mitochondrial swelling in isolated astrocytic mitochondria (Ziemińska et al., 2000a), in cultured astrocytes (Pichili et al., 2007) and in the brain of ALF-affected rats (Rama Rao et al., 2010).

In mammalian cells, mPTP elicits a rapid increase in the permeability of e.g., protons, ions, and other molecules of MW < 1,500 Da through the inner mitochondrial membrane. The mega-channels opening leads to a collapse of the mitochondrial inner membrane potential, created by protons through the mitochondrial electron transport chains (Guo et al., 2018; Fernandez-Vizarra and Zeviani, 2021). This causes osmotic swelling of the mitochondrial matrix and movement of metabolites through the inner membrane that leads to defective oxidative phosphorylation decrease of ATP synthesis (Vercellino and Sazanov, 2022). Consequently, the mitochondrial membrane potential loss leads to reactive oxygen species production that further stimulates the mPTP process.

In astrocytes, mitochondrial dysfunction elicited by glutamine leads to the formation of reactive oxygen species and astrocytic cell swelling (Norenberg et al., 2004; Albrecht and Norenberg, 2006; Rama Rao and Norenberg, 2012). Upon entry to mitochondria, glutamine is metabolized by the mitochondrial enzyme PAG to ammonia and glutamate and, ammonia is the trigger of subsequent deleterious events. This sequence of events has been documented in cultured astrocytes, where the glutamine-induced intra-astrocytic accumulation of reactive oxygen species (Jayakumar et al., 2004) and astrocytic swelling (Jayakumar et al., 2006) were attenuated, by the PAG synthetic inhibitor, DON (Crosby, 2015).

Evidence that served to formulate the “Trojan Horse” hypothesis has been considered somewhat ambiguous. The criticism raised was that the hypothesis was overwhelmingly based on results of *ex vivo* experiments (isolated mitochondria), or *in vitro* (cultured astrocytes treated with 5 mM ammonia for 24 h) (see the above paras). As a step forward, in one *in vivo* the mPTP induction was detected in brain homogenates of rats with thioacetamide-induced ALF, and reduced after the administration of glutamine transport competitor, histidine. Histidine also attenuated oxidative stress, and brain edema (Rama Rao et al., 2010). Toward the same end, intracellular lactate accumulation in the brain and elevated ICP occurred notwithstanding unchanged mitochondrial function in hyperammonemia brain tissue (Witt et al., 2017). In support of the “Trojan Horse” glutamine mode of action, elevated concentrations of ATP degradation products hypoxanthine and inosine, markers of mitochondrial dysfunction, in brain dialysates from ALF patients, have been found to correlate with extracellular glutamine (Bjerring et al., 2010). Less directly supportive of, but consistent with the hypothesis, reduced metabolic rates of oxygen were very previously noted in the brain of rats with thioacetamide-induced ALF (Pluta and Albrecht, 1986), and more recently in the brain of patients with overt hepatic encephalopathy (Iversen et al., 2009).

One other criticism of the Trojan Horse hypothesis has been raised based on the purported absence of PAG in native astrocytes and its exclusive location in neurons (Laake et al., 1999). In line with the discussed view, ameliorating effects of astrocyte swelling *in vitro*, induced by the PAG inhibitor, were interpreted as solely reflecting phenotypic alteration of astrocytes upon culturing. However, the skepticism was balanced by solid evidence that both PAG isoforms abound in the rat and human brain (Cardona et al., 2015; Milewski et al., 2019).

## Slc7A6: Contributor to Ammonia-Induced Oxidative/Nitrosative Stress in Astrocytic Swelling

Compelling evidence documents that ammonia generates free radicals (Murthy et al., 2001; Görg et al., 2008) and ammonia-induced astrocytic swelling that *per se* appears to be a trigger of oxidative/nitrosative stress and subsequent protein tyrosine nitration, is a critical event *en route* to astrocytic and neuronal dysfunction (Schliess et al., 2002; Kruczek et al., 2011). Induction of peroxynitrite activates volume-regulated anion channels in astrocytes and induces astrocytic swelling which was coupled to enhanced release of the glutamate surrogate [^3^H]D-aspartate (Haskew et al., 2002). Free radicals formation was observed in a rat model of hyperammonemia (Kosenko et al., 1997, 1998), and was associated with decreased activity of antioxidant enzymes (glutathione peroxidase, superoxide dismutase and catalase) (Kosenko et al., 1997). Ammonia-induced cerebrocortical tissue swelling was ameliorated by the NMDA/nitric oxide (NO) pathway inhibitors, indicating involvement of oxidative-nitrosative stress (Zielińska et al., 2003).

The system y + L member, Slc7a6 (4F2hc/y + LAT2) carrier is a heterodimer, composed of a glycosylated heavy chain (SLC3a2/4F2hc/CD98 and a catalytic light chain subunit (SLC7) (Torrents et al., 1998). The heavy chain is necessary for trafficking of the light chain to the plasma membrane, whereas the light chain determines the transport characteristics of the respective heterodimer. Slc7a6 has a wide tissue distribution including brain, heart, kidney, and promotes glutamine exchange for essential cationic amino acids including arginine (Pfeiffer et al., 1998; Torrents et al., 1998; Bröer et al., 2000).

The role of Slc7a6-mediated glutamine-arginine exchange in the modulation of NO synthesis under hyperammonemic conditions *in vivo* has been demonstrated in this laboratory (Hilgier et al., 2008, 2009). Specifically, in ammonia perfused rat brain, pharmacological inhibition of extracellular glutamine degradation or glutamine transport to neurons reduced NO synthesis and its accumulation in the extracellular space (Hilgier et al., 2009). Attenuation of NO synthesis was reproduced by intracerebral administration of glutamine, or two other y + LAT2 substrates, leucine and cyclo-leucine (Hilgier et al., 2009), while inhibition of glutamine synthesis reversed this attenuating effect (Hilgier et al., 2009). Collectively the results underscored the contribution of y + LAT2 transporter to glutamine/arginine imbalance associated with hyperammonemia. The link of y + LAT2 activity to ammonia-induced oxidative-nitrosative stress came to light in studies on cultured astrocytes and animal models (Zielińska et al., 2011, 2012, 2014). Those studies documented reduced arginine delivery due to enhanced y + LAT2-mediated exchange of extracellular glutamine for intracellular arginine, contributing to decreased NO and cGMP formation in the rat brain by hyperammonemia.

More recent studies revealed that induction of iNOS in astrocytes and stimulation of NO synthesis by ammonia is coupled to increased arginine uptake, a process promoted by a selective increase of the expression and activity of y + LAT2, whereas the unidirectional basic amino acid carrier y + was not affected by ammonia (Zielińska et al., 2012, 2015). In cultured astrocytes, ammonia-evoked oxidative-nitrosative stress also involves induction of iNOS (Schliess et al., 2002), mediated by activation of the nuclear factor κB (Sinke et al., 2008). Of note, cytotoxic brain edema in ALF is also related to the central or peripheral inflammation (Wilkinson et al., 1974; Wyke et al., 1982; Odeh et al., 2004; Shawcross et al., 2007; Butterworth, 2011; Scott, 2013; Jayakumar et al., 2015), whose deleterious effects are mediated by oxidative-nitrosative stress. However, specific contribution of glutamine transport to this phenomenon has to our knowledge not been investigated as yet.

## Concluding Remarks

**FIGURE 1:**
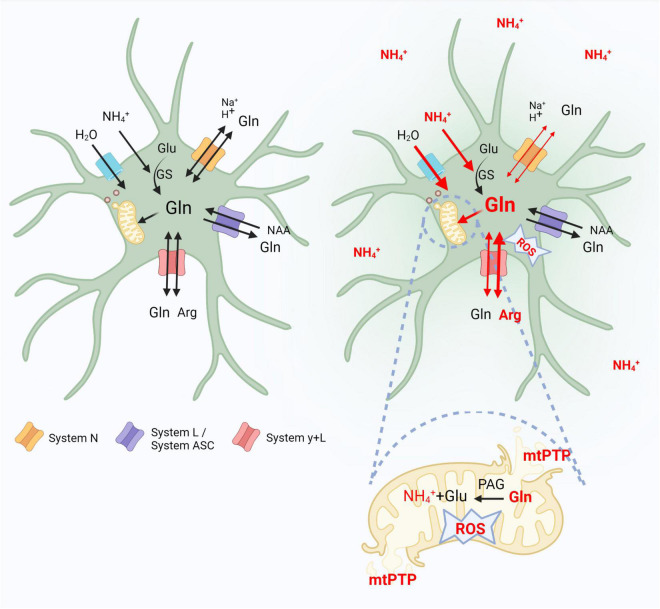
Contribution of astrocytic glutamine transporters belonging to systems N, L/ASC and y + L to the glutaminosis-driven aspect of brain edema in acute hyperammonemia. Left panel indicates control conditions. In the right, where the effects of hyperammonemia are outlined, thin or thick red lines indicate decreased or increased activity of a given event, respectively. Glu, glutamate; Gln, glutamine; Arg, arginine; GS, glutamine synthetase; NAA, neutral amino acids; ROS, reactive oxygen species; mtPTP, mitochondria permeability transition pore. Created with BioRender.com.

In conclusion, data presented in this review support the view that altered functioning of glutamine transporting moieties residing in astrocytes, contribute in a vicious circle mode, to the glutamine-driven aspect of astrocytic swelling and cytotoxic brain edema associated with ALF (Figure 1). Decreased Slc38a3 activity reduces efflux of newly synthesized glutamine, augmenting its intra-astrocytic residence. The resulting glutamine surplus aggravates glutamine-induced osmotic stress in the cytoplasm, at the same time rendering more glutamine available for (i) its Slc7a6-mediated exchange for extracellular arginine, which increases intra-astrocytic accumulation of reactive nitrogen species (ii) glutamine transport to astrocytic mitochondria, which leads to mitochondrial dysfunction (the “Trojan Horse” mode of action). The effects of (i) and (ii) converge at the level of oxidative/nitrosative stress, a critical driver of astrocytic swelling. Clearly, evidence for the above reasoning, especially regarding glutamine transport to mitochondria needs to be fine-tuned to achieve full symmetry between the documentation *in vivo* and *in vitro*.

In astrocytes, hyperammonemia (ammonium ions, NH_4_^+^) alters the functioning of glutamine transporters that contribute in a vicious circle mode to the glutamine-driven aspect of astrocytic swelling and cytotoxic brain edema. Decreased system N (Slc38a3) activity reduces efflux of synthesized by glutamine synthetase (GS) glutamine, augmenting its intra-astrocytic residence. The resulting glutamine surplus aggravates glutamine-induced osmotic stress in the cytoplasm, rendering more glutamine available for: (i) its system y + L (Slc7a6)-mediated exchange for extracellular arginine (Arg), which increases the intra-astrocytic accumulation of reactive oxygen-nitrogen species (ROS), (ii) glutamine enter to astrocytic mitochondria, where, hydrolyzed to glutamate and NH_4_^+^ by PAG leads to open mitochondrial permeability transition pore (mPTP), and mitochondrial dysfunction (the “Trojan Horse” mode of action).

## Author Contributions

MZ, JA, and MP wrote the manuscript. MZ and MP designed the figure, tables, and contributed to the revision of the manuscript. All authors contributed to the article and approved the submitted version.

## Conflict of Interest

The authors declare that the research was conducted in the absence of any commercial or financial relationships that could be construed as a potential conflict of interest.

## Publisher’s Note

All claims expressed in this article are solely those of the authors and do not necessarily represent those of their affiliated organizations, or those of the publisher, the editors and the reviewers. Any product that may be evaluated in this article, or claim that may be made by its manufacturer, is not guaranteed or endorsed by the publisher.
